# Delays in Coccidioidomycosis Diagnosis and Relationship to Healthcare Utilization, Phoenix, Arizona, USA^1^

**DOI:** 10.3201/eid2509.190019

**Published:** 2019-09

**Authors:** Rachel Ginn, Ralph Mohty, KeriLyn Bollmann, Jessica Goodsell, Guillermo Mendez, Barrie Bradley, John N. Galgiani

**Affiliations:** Banner Health Corporation, Phoenix, Arizona, USA (R. Ginn, J. Goodsell, G. Mendez, B. Bradley);; University of Arizona College of Medicine—Phoenix, Phoenix (R. Mohty, K. Bollmann, J.N. Galgiani);; Banner University Medical Center–Phoenix, Phoenix (K.L. Bollmann, J.N. Galgiani);; University of Arizona College of Medicine—Tucson, Tucson, Arizona, USA (J.N. Galgiani)

**Keywords:** population health, coccidioidomycosis, healthcare utilization, diagnosis, medical costs, fungi, Arizona, United States, Valley fever

## Abstract

We developed an electronic records methodology to programmatically estimate the date of first appearance of coccidioidomycosis symptoms in patients. We compared the diagnostic delay with overall healthcare utilization charges. Many patients (46%) had delays in diagnosis of >1 month. Billed healthcare charges before diagnosis increased with length of delay.

Coccidioidomycosis (also known as Valley fever) is an endemic fungal infection (*1*,*2*). In Arizona, USA, it is responsible for one quarter of all community-acquired pneumonia (CAP) (*3*,*4*). Although accurate diagnosis requires specific laboratory tests (*5*), such testing was only ordered in <13% of patients with CAP in Phoenix, Arizona (*6*), raising the possibility that delays in accurate diagnosis might be extensive. We report a retrospective analysis from the coccidioidomycosis-endemic region of Phoenix to estimate diagnostic delays and healthcare utilization before and after a coccidioidal diagnosis was confirmed.

## The Study

This study focused on cases recorded at clinics operated by Banner Health in the Phoenix area, using programmatic use of data from NextGen (https://www.nextgen.com). This electronic medical record system was used during the study period by most of Banner Health’s clinics but not its hospitals. We programmatically searched the system for all patients >18 years of age who were seen from January 1, 2011, through December 31, 2014, whose records showed codes from the International Classification of Diseases, 9th Revision (ICD-9), that indicated coccidioidomycosis (114.*). The earliest diagnosis of coccidioidomycosis served as the diagnosis date for each patient. We excluded patients with a coccidioidomycosis diagnosis in 2011 who also had a coccidioidomycosis diagnosis in the prior 12 months. We corroborated all diagnoses by a programmatic identification of any positive coccidioidal serologic test result (*5*) <30 days before or >60 days after the date of diagnosis.

To estimate the date when patients first sought medical attention for their illness, we used a set of ICD-9 codes that represented an expansion of codes previously associated with early coccidioidal illnesses that were used in a Phase I independent chart review of 37 patients performed by 2 physicians in training and an internist (*7*). For our study, an internist and a medical student independently reviewed all ICD-9 codes occurring 6 months before the coccidioidomycosis diagnosis and included only those that they both judged to be similar to the original set from the previous Phase I study. An infectious disease specialist settled cases of nonconcordance. We removed 3 Phase I symptoms that were deemed erroneous. This process resulted in a total of 121 ICD-9 codes (Appendix Table). We defined the date of first presentation (i.e., the date signs and symptoms were first clinically noted) as the earliest record in which >1 of the expanded ICD-9 codes were recorded, within 6 months before the diagnosis date. We calculated the delay in diagnosis as the difference between the date of first presentation and the diagnosis date.

Healthcare charges represented total healthcare utilization. Billing data were available for ambulatory care from NextGen and for Banner Health’s hospitalizations from MedSeries4 (Siemens Medical Solutions USA, Inc., https://www.siemens.com). We defined healthcare utilization as the total charges, for any cause, from the date of first visit to 6 months after the diagnosis date. We distinguished total charges from actual reimbursement amount because the total is only 1 aspect of a complicated financial billing system.

We used the SAS Enterprise Guide Software Suite version 7.13 (SAS Institute Inc., https://www.sas.com) for data retrieval and analysis. The Banner Health Institutional Review Board approved this study.

We identified newly diagnosed coccidioidal infections in 139 patients. The mean patient age was 49.2 years (SD + 15.3 years) (Appendix Figure 1), and 52% of patients were female.

The 3 most frequently occurring coccidioidal ICD-9 codes were unspecified (114.9, 47%), primary pulmonary (114.0, 37%), and pulmonary unspecified (114.5, 8%) (Appendix Figure 2). Confirmatory serologic tests were recorded within 15 days before or after the date of diagnosis in 81% of patients (Appendix Figure 3).

The expanded set of ICD-9 codes failed to identify an initial presentation date for 20 patients. Of the 119 patients with an identified presentation date, 16 (13%) received a diagnosis on the day of presentation, 24% within 1 week of first presentation, 46% within 1 month, and 54% from 30 days to 6 months after presentation (Figure).

**Figure:**
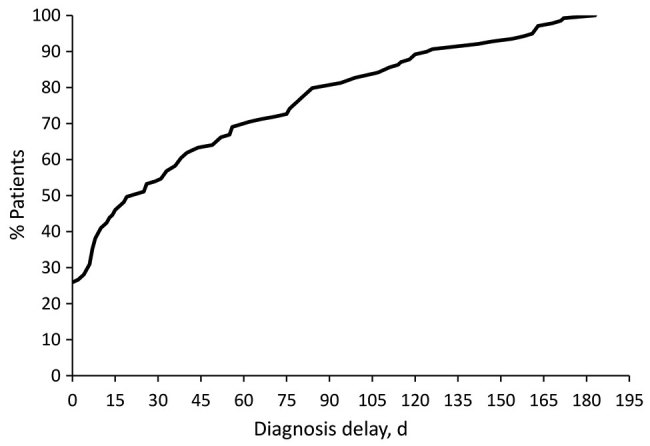
Cumulative distribution of coccidioidomycosis patient population in relation to diagnosis delay for cohort of patients in Phoenix, Arizona, USA. At 30 days of delay, ≈55% of the population had received a diagnosis.

We queried total charges for all 139 patients from the date of first visit (the coccidioidomycosis diagnosis date, if no initial presentation date was available) until 6 months after the diagnosis date. By cross-referencing patient identifiers, we found 66 (47%) patients to have hospitalization billings, which were included in the total healthcare cost. Median healthcare charges seemed to increase as diagnosis delays increased (Table). When we compared diagnosis delay to the log-normal of total charges, we found a significant, positive, linear relationship for overall charges (R^2^ = 0.07, p = 0.003), attributable to those occurring before (R^2^ = 0.16, p<0.0001) but not to those on or after the diagnosis date (R^2^ = 0.01, p = 0.2748). Although we saw issues with data normality because of our small sample size, we assumed normality for linear regression analysis (Appendix Figures 4–6).

**Table:** Median charges by monthly range of diagnostic delays before and after the date of coccidioidomycosis diagnosis for cohort of patients in Phoenix, Arizona, USA*

Time from presentation to diagnosis, mo	No. patients	Before diagnosis			After diagnosis			Combined	
		Median	Total		Median	Total		Median	Total
<1	55	$373	$30,081		$637	$239,681		$749	$269,762
1–3	37	$791	$217,662		$2,707	$436,233		$2,492	$653,895
4–6	27	$915	$338,810		$1,199	$710,911		$2,680	$1,049,720
No recorded symptoms	20	NA	NA		$4,172	$854,597		$4,172	$854,597
Overall	139	$637	$586,553		$875	$2,241,422		$1,231	$2,827,974

*NA, not applicable.

## Conclusions

For this retrospective study, we developed a set of ICD-9 codes to use as a surrogate indicator for the date coccidioidomycosis patients were first seen within Banner Health clinics for signs or symptoms of their infection. This list expanded on an initial set of codes discovered through a manual chart review (*7*). Many of the codes are common and are unlikely themselves to be predictive of diagnosis. Previous studies have been unable to meaningfully distinguish presenting syndromes of coccidioidomycosis in patients from other overlapping etiologies (*3*,*4*,*8*). However, this code set was useful to computationally establish the date of first healthcare contact for these patients. If this analytic method is validated and perhaps improved in future studies, it could allow for automated monitoring of diagnostic delay as a metric of quality assessment, without the need for expert chart review.

Of the 139 patients analyzed in this study, 20 did not have an eligible ICD-9 code, and 16 others had their initial presentation code recorded on the same day as their coccidioidomycosis diagnosis. Factors leading to these problems might include failure of a clinician to record sufficient diagnostic information or use of codes that are not sufficiently comprehensive. Future studies could manually examine the full patient records to further expand the code set. It is also possible that patients entered the Banner Health electronic system with a diagnosis of coccidioidomycosis from elsewhere or that the coccidioidal infection was truly without symptoms.

This investigation identified a prolonged diagnosis delay for much of the population; 46% had a delay >1 month (Figure). With increased delays, total healthcare charges increased substantially. However, this study was not limited to coccidioidomycosis-related charges; any diagnosis delay could result in increased healthcare utilization, regardless of relationship to coccidioidomycosis. In addition, data gaps may have occurred because of difficulty in cross-referencing identifiers or visits by patients to a non-Banner facility. However, our findings are consistent with the simple concept that diagnostic delays in a protracted coccidioidal illness would result in additional testing, visits, and possible use of empiric antibacterial treatments until a precise diagnosis is achieved.

Our study investigates only patients with confirmed coccidioidomycosis and not patients who never received a correct diagnosis. Previous studies indicate that appropriate testing for coccidioidomycosis is often not done in Phoenix and Tucson (*6*,*9*,*10*). Thus, the costs of delayed coccidioidal diagnosis probably underestimates the situation for this clinical practice within a coccidioidomycosis-endemic region. A recent study of coccidioidomycosis diagnosed outside endemic regions also showed substantial delays (*11*), making our research relevant to those with a recent travel history to endemic regions.

Appendix Additional information about coccidioidomycosis diagnosis delay and effects on healthcare utilization, Arizona, United States.

## References

[1] Hector RF, Rutherford GW, Tsang CA, Erhart LM, McCotter O, Anderson SM, et al. The public health impact of coccidioidomycosis in Arizona and California. Int J Environ Res Public Health. 2011;8:1150–73. 10.3390/ijerph8041150 21695034 PMC3118883

[2] Litvintseva AP, Marsden-Haug N, Hurst S, Hill H, Gade L, Driebe EM, et al. Valley fever: finding new places for an old disease: *Coccidioides immitis* found in Washington State soil associated with recent human infection. Clin Infect Dis. 2015;60:e1–3. 10.1093/cid/ciu681 25165087 PMC4296125

[3] Valdivia L, Nix D, Wright M, Lindberg E, Fagan T, Lieberman D, et al. Coccidioidomycosis as a common cause of community-acquired pneumonia. Emerg Infect Dis. 2006;12:958–62. 10.3201/eid1206.060028 16707052 PMC3373055

[4] Kim MM, Blair JE, Carey EJ, Wu Q, Smilack JD. Coccidioidal pneumonia, Phoenix, Arizona, USA, 2000-2004. Emerg Infect Dis. 2009;15:397–401. 10.3201/eid1563.081007 19239751 PMC2681119

[5] Galgiani JN, Ampel NM, Blair JE, Catanzaro A, Geertsma F, Hoover SE, et al. 2016 Infectious Diseases Society of America (IDSA) clinical practice guideline for the treatment of coccidioidomycosis. Clin Infect Dis. 2016;63:e112–46. 10.1093/cid/ciw360 27470238

[6] Chang DC, Anderson S, Wannemuehler K, Engelthaler DM, Erhart L, Sunenshine RH, et al. Testing for coccidioidomycosis among patients with community-acquired pneumonia. Emerg Infect Dis. 2008;14:1053–9. 10.3201/eid1407.070832 18598625 PMC2600364

[7] Bollmann K, Narasimhan S, Crawford N, Fields N, Yazdchi D, Goodsell J, et al. Rapidity of coccidioidomycosis diagnosis and its effect on healthcare utilization [poster presentation]. In: American College of Physicians Arizona Chapter 2015 Annual Meeting; November 13–15, 2015; Tucson, Arizona, USA [cited 2019 Jun 17]. https://www.acponline.org/system/files/documents/about_acp/chapters/az/salud_15.pdf

[8] Yozwiak ML, Lundergan LL, Kerrick SS, Galgiani JN. Symptoms and routine laboratory abnormalities associated with coccidioidomycosis. West J Med. 1988;149:419–21. 3227686 PMC1026485

[9] Campion JM, Gardner M, Galgiani JN. Coccidioidomycosis (Valley fever) in older adults: an increasing problem. Ariz Geriatr Soc J. 2003;8:3–12.

[10] Stern NG, Galgiani JN. Coccidioidomycosis among scholarship athletes and other college students, Arizona, USA. Emerg Infect Dis. 2010;16:321–3. 10.3201/eid1602.090918 20113571 PMC2958011

[11] Benedict K, Ireland M, Weinberg MP, Gruninger RJ, Weigand J, Chen L, et al. Enhanced surveillance for coccidioidomycosis, 14 US states, 2016. Emerg Infect Dis. 2018;24:1444–52. 10.3201/eid2408.171595 30014837 PMC6056093

