# Supplementary material for: Delays in Coccidioidomycosis Diagnosis and Relationship to Healthcare Utilization, Phoenix, Arizona, USA1
Source: Emerg Infect Dis. 2019 Sep;25(9):1742–4. doi: 10.3201/eid2509.190019 (PMC6711213; doi:10.3201/eid2509.190019)
Supplement: Appendix — Additional information about coccidioidomycosis diagnosis delay and effects on healthcare utilization, Arizona, United States. [file 19-0019-Techapp-s1.pdf]

Article DOI: https://doi.org/10.3201/eid2509.190019

Delays in Coccidioidomycosis Diagnosis and Relationship to Healthcare Utilization, Arizona, USA

Appendix

Appendix Table. Expanded ICD-9 code list used to define the initial presentation date in study of delays in coccidioidomycosis diagnosis.

ICD-9 code	Diagnosis	Phase I symptoms*	Phase II expansion†	No. initial symptoms‡	No. total symptoms§
780.79	Malaise and fatigue	Yes	Yes	29	61
786.2	Cough	Yes	Yes	27	86
486	Pneumonia	No	Yes	10	43
493.90	Unspecified asthma, uncomplicated	No	Yes	8	13
692.9	Unspecified contact dermatitis, unspecified cause	No	Yes	7	11
782.1	Rash and nonspecific skin eruption	Yes	Yes	7	13
465.9	Acute upper respiratory infection, unspecified	No	Yes	6	9
477.9	Allergic rhinitis, unspecified	No	Yes	5	11
724.2	Low back pain	No	Yes	5	6
782.3	Edema, unspecified	No	Yes	5	19
V79.0	Encounter for screening for other disorder	No	Yes	4	4
461.9	Acute sinusitis, unspecified	No	Yes	3	7
473.9	Unspecified sinusitis (chronic)	Yes	Yes	3	3
719.41	Pain in unspecified shoulder	No	Yes	3	8
780.60	Fever, unspecified	Yes	Yes	3	10
784.0	Headache (facial pain)	Yes	Yes	3	6
786.50	Unspecified chest pain	Yes	Yes	3	12
079.99	Other viral agents as the cause of diseases classified elsewhere	No	Yes	2	3
415.19	Other pulmonary embolism without acute cor pulmonale	No	Yes	2	18
462	Acute pharyngitis	Yes	Yes	2	4
466.0	Bronchitis	No	Yes	2	8
490	Bronchitis	No	Yes	2	5
496	Chronic obstructive pulmonary disease, unspecified	No	Yes	2	7
708.9	Urticaria, unspecified	No	Yes	2	4
714.9	Inflammatory polyarthropathy	No	Yes	2	7
719.00	Effusion, unspecified joint	Yes	Yes	2	3
719.40	Pain in unspecified joint	Yes	Yes	2	4
719.43	Pain in unspecified wrist	No	Yes	2	3
719.47	Pain in unspecified ankle and joints of unspecified foot	No	Yes	2	3
719.49	Pain in unspecified joint	No	Yes	2	5
722.4	Other cervical disc degeneration, unspecified cervical region	No	Yes	2	6
724.5	Dorsalgia, unspecified	No	Yes	2	4
729.1	Myositis, unspecified	No	Yes	2	6
733.90	Disorder of bone, unspecified	No	Yes	2	6
780.4	Dizziness and giddiness	No	Yes	2	3
786.05	Shortness of breath	No	Yes	2	13
786.52	Chest pain on breathing	No	Yes	2	2
786.59	Chest pain	Yes	Yes	2	4
V12.09	Personal history of other infectious and parasitic diseases	No	Yes	2	3
288.60	Elevated leukocyte count, unspecified	No	Yes	1	2
382.9	Otitis media, unspecified, unspecified ear	No	Yes	1	1

ICD-9 code	Diagnosis	Phase I symptoms*	Phase II expansion†	No. initial symptoms‡	No. total symptoms§
461.0	Acute maxillary sinusitis, unspecified	No	Yes	1	3
461.8	Acute pansinusitis, unspecified	No	Yes	1	1
477.8	Other allergic rhinitis	No	Yes	1	3
482.89	Pneumonia	No	Yes	1	2
511.0	Pleural condition, unspecified	No	Yes	1	3
518.89	Other diseases of lung	Yes	Yes	1	3
695.2	Erythema nodosum	No	Yes	1	6
698.9	Pruritus, unspecified	No	Yes	1	1
709.9	Disorder of the skin and subcutaneous tissue, unspecified	No	Yes	1	6
719.07	Effusion, unspecified ankle	No	Yes	1	2
719.42	Pain in unspecified elbow	No	Yes	1	1
719.46	Pain in unspecified knee	No	Yes	1	2
722.93	Discitis, unspecified, lumbosacral region	No	Yes	1	1
723.1	Cervicalgia	No	Yes	1	2
729.5	Pain in unspecified limb	No	Yes	1	7
733.6	Chondrocostal junction syndrome [Tietze]	No	Yes	1	5
739.2	Segmental and somatic dysfunction of thoracic region	No	Yes	1	2
780.99	Other general symptoms and signs	No	Yes	1	2
782.2	Localized swelling, mass and lump, unspecified	No	Yes	1	3
785.6	Enlarged lymph nodes, unspecified	No	Yes	1	5
786.07	Wheezing	No	Yes	1	1
786.09	Dyspnea and respiratory abnormality	Yes	Yes	1	5
995.3	Allergy, unspecified, initial encounter	No	Yes	1	1
V74.1	Encounter for screening for respiratory tuberculosis	No	Yes	1	4
057.9	Unspecified viral infection characterized by skin and mucous membrane lesions	No	Yes	0	1
288.3	Eosinophilia	Yes	No	0	0
289.3	Nonspecific lymphadenitis, unspecified	No	Yes	0	1
307.81	Tension-type headache, unspecified, not intractable	No	Yes	0	1
478.19	Other specified disorders of nose and nasal sinuses	No	Yes	0	2
478.70	Other diseases of larynx	No	Yes	0	3
480.0	Pneumonia	No	No	0	0
480.1	Pneumonia	No	No	0	0
480.2	Pneumonia	No	No	0	0
480.3	Pneumonia	No	No	0	0
480.8	Pneumonia	No	No	0	0
481	Pneumonia	No	No	0	0
482.0	Pneumonia	No	No	0	0
482.1	Pneumonia	No	No	0	0
482.2	Pneumonia	No	No	0	0
482.32	Pneumonia	No	No	0	0
482.39	Pneumonia	No	No	0	0
482.40	Pneumonia	No	No	0	0
482.41	Pneumonia	No	No	0	0
482.42	Pneumonia	No	No	0	0
482.49	Pneumonia	No	No	0	0
482.82	Pneumonia	No	No	0	0
482.83	Pneumonia	No	No	0	0
482.9	Pneumonia	No	Yes	0	1
483.0	Pneumonia	No	No	0	0
483.1	Pneumonia	No	No	0	0
483.8	Pneumonia	No	No	0	0
484.7	Pneumonia	No	No	0	0
485	Pneumonia	No	No	0	0
487.0	Pneumonia	No	No	0	0
487.1	Pneumonia	No	No	0	0
487.1	Bronchitis	No	No	0	0
491.0	Bronchitis	No	No	0	0
491.1	Bronchitis	No	No	0	0
491.8	Bronchitis	No	No	0	0
491.9	Bronchitis	No	No	0	0

ICD-9 code	Diagnosis	Phase I symptoms*	Phase II expansion†	No. initial symptoms‡	No. total symptoms§
493.92	Unspecified asthma with (acute) exacerbation	No	Yes	0	1
514	Pneumonia	No	No	0	0
695.11	Other erythema multiforme	No	Yes	0	1
708.0	Allergic urticaria	No	Yes	0	1
719.45	Pain in unspecified hip	No	Yes	0	2
724.1	Pain in thoracic spine	No	Yes	0	1
729.89	Other symptoms and signs involving the musculoskeletal system	No	Yes	0	1
780.64	Chills (without fever)	No	Yes	0	1
780.8	Generalized hyperhidrosis	Yes	Yes	0	2
780.96	Pain, unspecified	Yes	No	0	0
784.2	Localized swelling, mass and lump, head	No	Yes	0	1
786.02	Orthopnea	No	Yes	0	1
786.30	Hemoptysis, unspecified	Yes	Yes	0	1
786.6	Localized swelling, mass and lump, trunk	No	Yes	0	6
786.9	Other abnormalities of breathing	No	Yes	0	1
793.11	Nonspecific abnormal findings on radiological/other examination of solitary pulmonary nodule	Yes	Yes	0	6
793.19	Other nonspecific abnormal finding of lung field	No	Yes	0	10
796.4	Other general symptoms and signs	No	Yes	0	2
807.00	Closed fracture, unspecified rib(s)	Yes	No	0	0
829.0	Fracture of unspecified bones, closed	Yes	No	0	0

*These ICD-9 codes originated from the Phase I chart review. These are subject to the study design and conditions therein.
†Expanded Phase II symptoms are based on 6-month diagnosis history, before the diagnosis dates for coccidioidomycosis. These were determined to be similar to the Phase I symptoms by the review process described in the article. Thus, they are dependent on the results of the Phase I study, but served to provide a wider array of ICD-9 codes to capture the Phase I concepts.
‡Includes the ICD-9 codes that were found on the earliest symptom date for the population, 6 months before the date of diagnosis for coccidioidomycosis. The earliest symptom date was used to calculate the diagnosis delay in conjunction with the diagnosis date. It is possible for 1 patient to have multiple symptoms on a day, which would increase the total aggregate of these values.
§Total number of symptom diagnoses 6 months before coccidioidomycosis diagnosis dates for the population. Only a fraction of these served as the initial symptom.

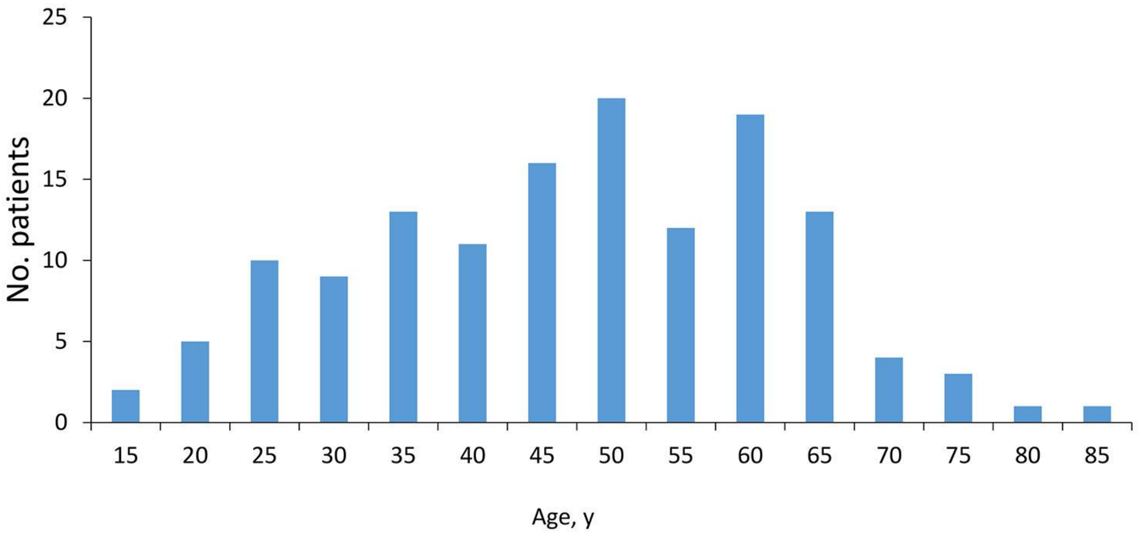

Appendix Figure 1. Age distribution of the population of a study of delayed coccidioidomycosis diagnosis, Arizona, USA.

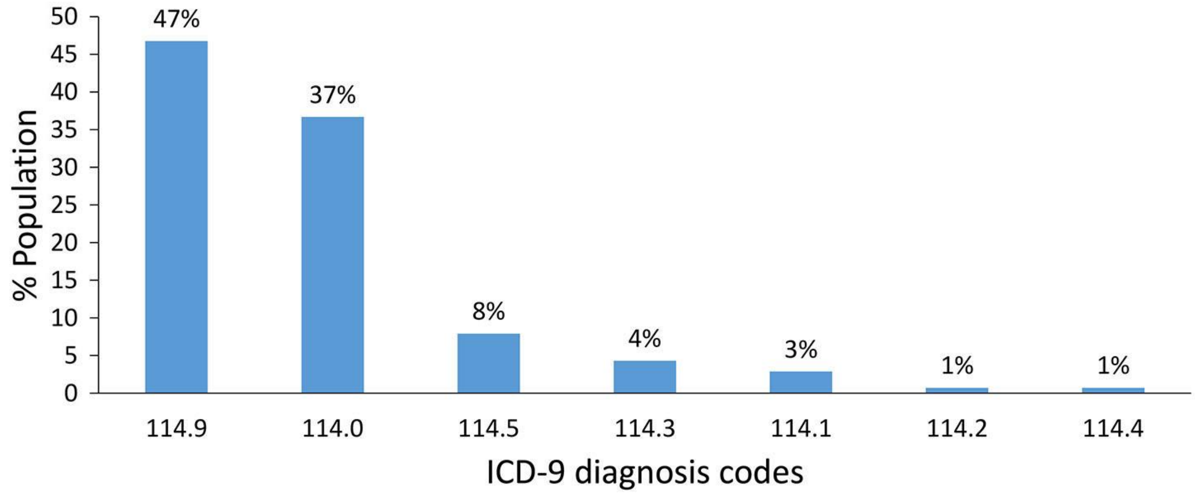

Appendix Figure 2. Distribution of diagnosis codes used to identify date of coccidioidomycosis diagnosis in patients in Arizona, USA.

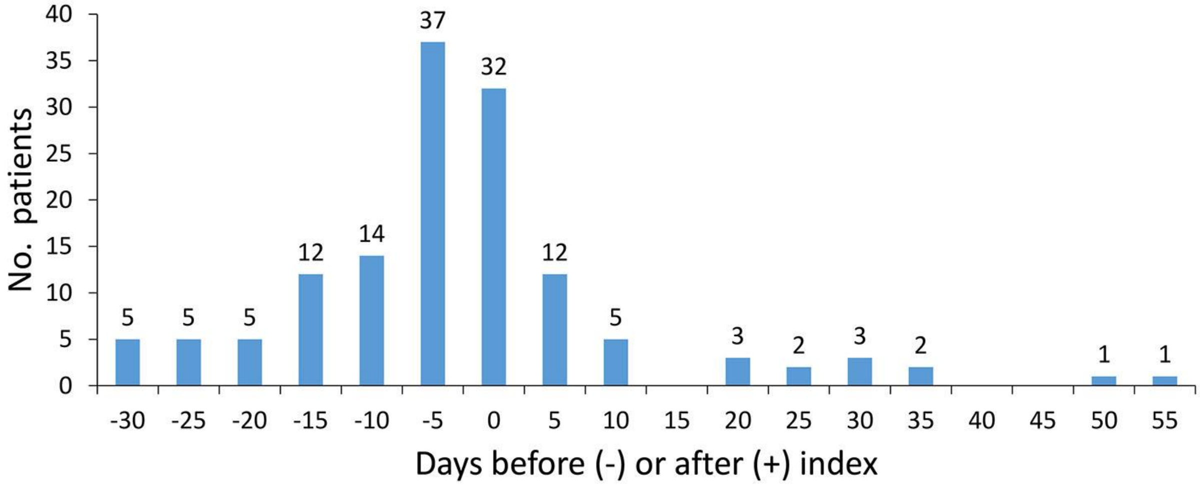

Appendix Figure 3. The time difference in days between the serology confirmation date and date of coccidioidomycosis diagnosis (index date), Arizona, USA.

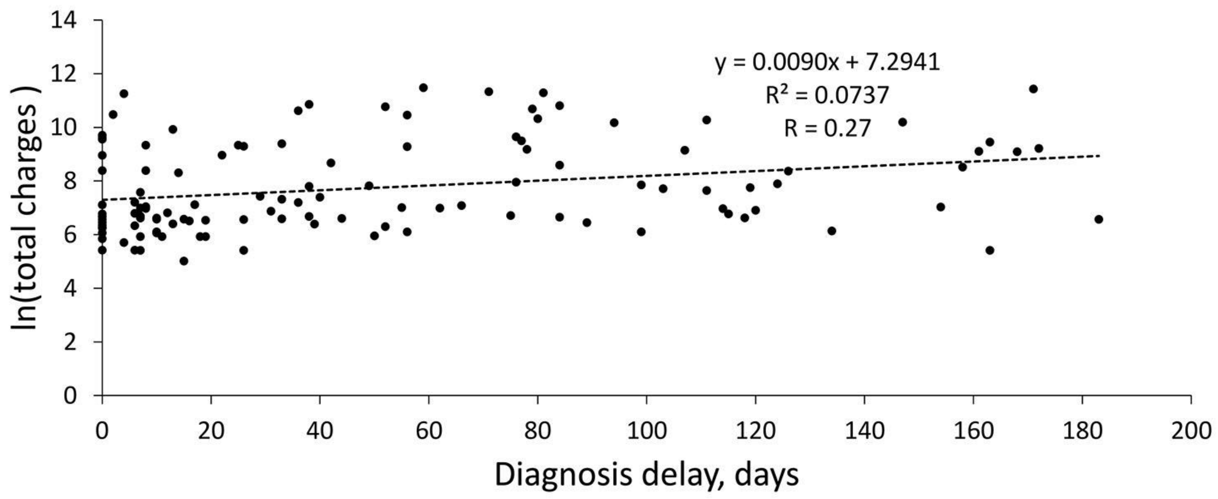

Appendix Figure 4. Linear regression model, assuming normality, for diagnosis delay vs. the lognormal (ln) of total healthcare charges for patients with coccidioidomycosis diagnosis, Arizona, USA. Only overall total charges are shown. The dashed line represents the fitted linear regression model.

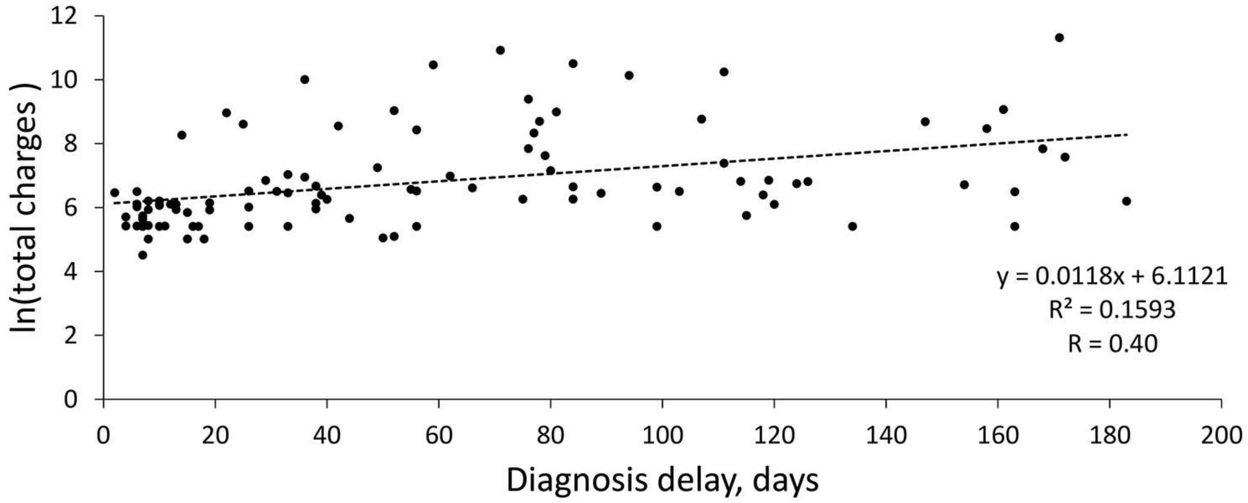

Appendix Figure 5. Linear regression model, assuming normality, for diagnosis delay vs. the lognormal (ln) of total healthcare charges for patients with coccidioidomycosis diagnosis, Arizona, USA. Only total charges before coccidioidomycosis diagnosis are shown. The dashed line represents the fitted linear regression model.

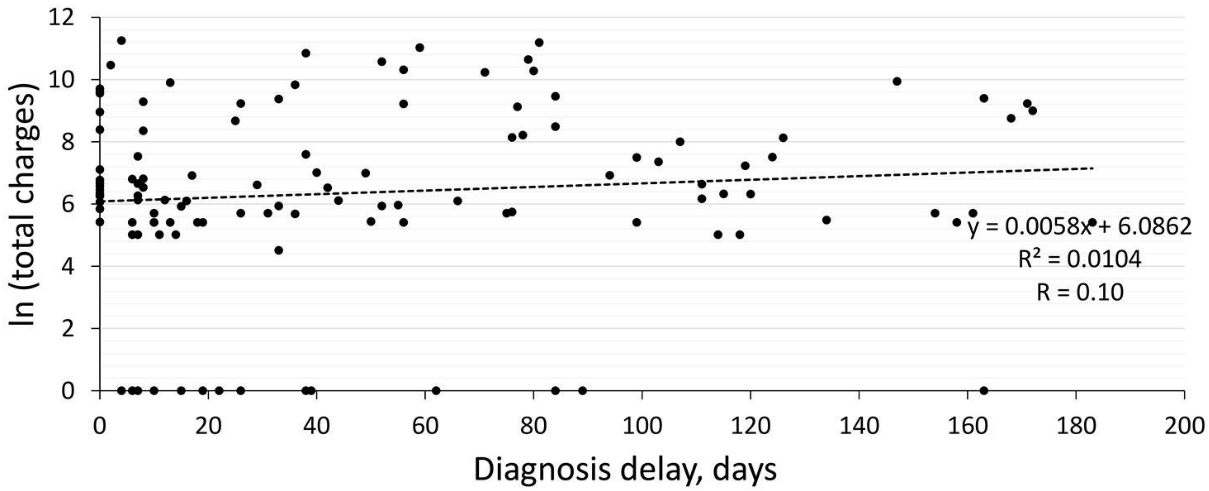

Appendix Figure 6. Linear regression model, assuming normality, for the diagnosis delay vs. the lognormal (ln) of total healthcare charges for patients with coccidioidomycosis diagnosis, Arizona, USA. Only total charges on or after CM diagnosis are shown. The dashed line represents the fitted linear regression model.
